# Regulation of Mesenchymal Stem Cell Differentiation by Nanopatterning of Bulk Metallic Glass

**DOI:** 10.1038/s41598-018-27098-6

**Published:** 2018-06-08

**Authors:** Ayomiposi M. Loye, Emily R. Kinser, Sabrine Bensouda, Mahdis Shayan, Rose Davis, Rui Wang, Zheng Chen, Udo D. Schwarz, Jan Schroers, Themis R. Kyriakides

**Affiliations:** 1 Center for Research on Interface Structures and Phenomena, Yale University, New Haven, CT 06520 USA; 2 Department of Biomedical Engineering, Yale University, New Haven, CT 06520 USA; 3 Department of Mechanical Engineering and Materials Science, Yale University, New Haven, CT 06520 USA; 4 IBM Thomas J, Watson Research Center, New York, NY 10598 USA; 5 Department of Molecular, Cellular and Developmental Biology, Yale University, New Haven, CT 06520 USA; 6 Department of Chemical and Enviromental Engineering, Yale University, P.O. Box 208089, New Haven, CT 06520 USA; 7 Department of Pathology, Yale University, P.O. Box 208089, New Haven, CT 06520 USA

## Abstract

Mesenchymal stem cell (MSC) differentiation is regulated by surface modification including texturing, which is applied to materials to enhance tissue integration. Here, we used Pt_57.5_Cu_14.7_Ni_5.3_P_22.5_ bulk metallic glass (Pt-BMG) with nanopatterned surfaces achieved by thermoplastic forming to influence differentiation of human MSCs. Pt-BMGs are a unique class of amorphous metals with high strength, elasticity, corrosion resistance, and an unusual plastic-like processability. It was found that flat and nanopattened Pt-BMGs induced osteogenic and adipogenic differentiation, respectively. In addition, osteogenic differentiation on flat BMG exceeded that observed on medical grade titanium and was associated with increased formation of focal adhesions and YAP nuclear localization. In contrast, cells on nanopatterned BMGs exhibited rounded morphology, formed less focal adhesions and had mostly cytoplasmic YAP. These changes were preserved on nanopatterns made of nanorods with increased stiffness due to shorter aspect ratios, suggesting that MSC differentiation was primarily influenced by topography. These observations indicate that both elemental composition and nanotopography can modulate biochemical cues and influence MSCs. Moreover, the processability and highly tunable nature of Pt-BMGs enables the creation of a wide range of surface topographies that can be reproducibly and systematically studied, leading to the development of implants capable of engineering MSC functions.

## Introduction

Mesenchymal stem cells (MSC)s are multipotent progenitor cells with useful properties for regenerative medicine by affecting inflammation, vascularization, and overall tissue regeneration^1^. The ability of MSCs to improve regeneration in a wound microenvironment is mediated by their differentiation into parenchymal cells and the production of growth factors^2^. Current tissue engineering approaches utilize structural scaffolds as mechanically stable MSC carriers that influence differentiation to reconstruct hard and soft tissues. Adult tissue, such as bone marrow, adipose tissue, placenta, amniotic fluid, and dental pulp, serve as wealthy depots of MSCs that have the capacity to proliferate and differentiate into mature specialized cells under favorable conditions^3,4^. Additionally, these cells, which are capable of at least tri-lineage differentiation into osteoblasts, adipocytes and chondroblasts, are less prone to tumor formation and allogeneic rejection in comparison to embryonic stem cells (ES)^5^. Clinically, MSCs have been transplanted into patients to treat osteogenesis imperfecta, Crohn’s diseases, and graft versus host disease (GVHD)^6^. MSCs have been shown to stimulate resident cells through paracrine signaling and matrix remodeling to enhance the differentiation of progenitor cells^7^. These desirable properties suggest that MSCs can be used together with biomaterials for enhanced regeneration. Biomaterials such as metallic alloys, used in conjunction with MSCs, provide a microenvironment for biomechanical, structural, and cellular support to promote tissue regeneration in orthopaedic applications due to their stability and low immunogenicity^1^. Furthermore, these materials can be modified to create *in vitro* niches with diverse biophysical and biochemical properties for investigating stem cell differentiation and function^3^.

Traditional metals and metallic alloys are limited by complex fabrication procedures, the use of toxic solvents, lack of bioactivity, and lack of long term stability in corrosive environments^8^. Platinum BMGs (Pt-BMG) are a novel class of materials with elastic moduli and yield strengths greater than traditionally available biomaterials, which allow for higher load capacities without deformation^9,10^. On an atomic scale, these materials lack crystalline order, grain boundaries, dislocations, and slip planes and are anisotropic while being homogenous, which allow for higher load capacities without deformation. Such structure results in amorphous bulk metals with high strength and elasticity, moderate Young’s moduli, and higher corrosion resistance than crystalline counterparts^11,12^. Unlike conventional metals and alloys such as titanium and stainless steel that must be heated past their melting temperature, Pt-BMGs can be processed like plastics in the supercooled region above their glass transition temperatures^13^. As a result, the amorphous structure of Pt-BMGs is not limited by an intrinsic size such as the grain size in crystalline metals or the chain length in polymers. Hence, Pt-BMGs can be molded on the micro and nano length scale with precision and high aspect ratios^14–16^. Furthermore, our previous work has shown biocompatibility of Pt-BMGs *in vivo* and *in vitro*^17,18^. Facile creation of accurate nanotopography on these surfaces enables the critical study of micro-environmental cues at a subcellular scale while revealing the effect of distinct biophysical cues on cell morphology, proliferation, and differentiation. This methodology informs the field of material science on the creation of modified materials for specific biomedical applications^19^.

Previous work has shown the role of biomechanics in directing the differentiation of MSCs into specific lineages (Table 1)^20–34^. Specifically, prior studies suggest that substrates that mimic the consistency of native tissues are ideal for engineering soft tissues, while connective tissue engineering is best on materials with high mechanical strength^35^. However, the interpretation of these results is complicated by the fact that most of the studies cited herein investigate stem cell differentiation on soft materials in the pascal to kilopascal range, versus the gigapascal range of metals. Additionally, while most studies assay nanotopography for bone engineering^36,37^, the effect of metallic, nanotopographical features on adipogenesis has not been investigated. To gain insight into the role of a high elastic moduli material on stem cell behavior, we investigated the effect of Pt-BMGs on human MSC proliferation, morphology, differentiation, and signaling. We chose grade 5 titanium (TiAl_6_V_4_) as a control due to its application in many load-bearing surgical interventions as a result of a high strength to weight ratio, biocompatibility, and a native oxide layer conducive to cell adherence^38^. It should be noted that in spite of these many favorable properties, titanium is a relatively bio-inert metal that requires excessive functionalization with hydroxyapatite, texturing, and the addition of growth factors to encourage osteoinduction via recruitment and differentiation of MSCs^39,40^.

**Table 1:** Elastic Moduli of Materials Currently Used in MSC Differentiation.

Class	Modulus	References
Natural Scaffolds	0.7 kPa–18 MPa	19–26
Polymers	16 kPa–1.5 GPa	27–31
Ceramics	72.9 GPa	32, 33

Here, we demonstrate that composition and topography of a material influence MSC responses and overcome the limitations of titanium. Specifically, we illustrate that in standard culture conditions, modulation of surface topography via nanopatterning caused a decrease in MSC size while inducing differentiation into an adipogenic lineage. Differentiation resulting in efficient adipogenesis on a material with elastic modulus in the GPa range is unexpected and has not been reported. Moreover, our findings suggest that Pt-BMG could be considered as an alternative to titanium, as evidenced by increased osteogenic differentiation under the same culture conditions. We also show that by changing composition and topography, we can significantly improve differentiation and alter mechanotransduction through YAP and vinculin subcellular distribution. Furthermore, we demonstrate that Pt-BMG substrates are efficient at improving adipogenic and osteogenic differentiation of MSCs. This work suggests the suitability of Pt-BMGs with surface modifications as functional scaffolds for tissue engineering applications *in vivo* and as a useful platform to study stem cell differentiation and signaling *in vitro*.

## Results

### Surface characterization

**Figure 1:**
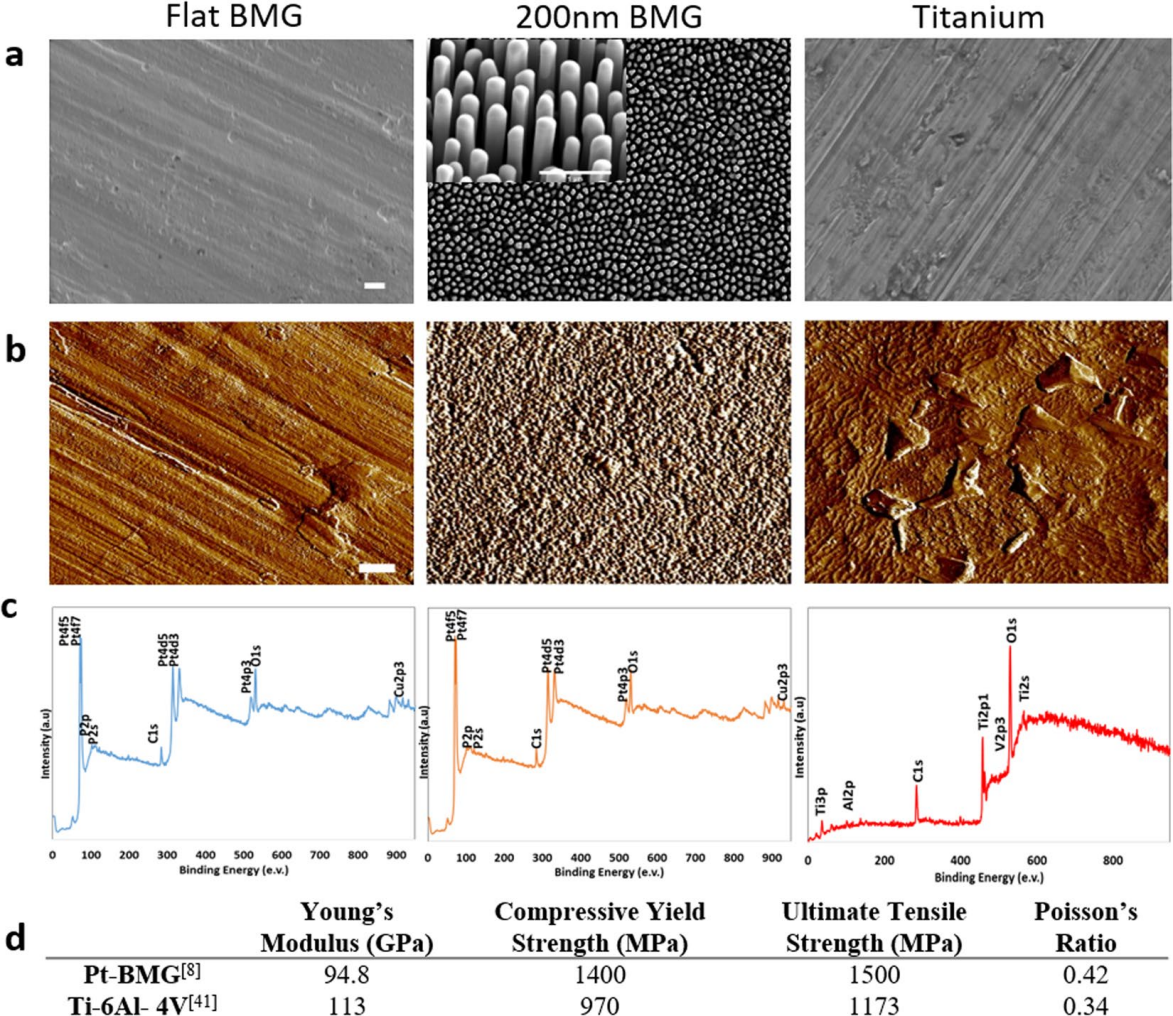
Characterization of substrates (**a**) Representative SEM images of Pt-BMG and titanium, scale bar = 1 μm. (**b**) Representative AFM images, scale bar = 1 μm. (**c**) Representative XPS Spectra. (**d**) Comparison of previously reported bulk mechanical properties of substrates.

We characterized substrates and confirmed specific topographies for the flat Pt-BMG, nanopatterned Pt-BMG, and titanium using scanning electron microscopy (SEM) and atomic force microscopy (AFM) (Fig. 1a,b). A 200 nm nominal diameter was chosen based on work done by Padmanabhan *et al*. that showed significant differences in cell morphology for various cell types on Pt-BMG. We fabricated the nanopatterned surface with nanorods of a nominal diameter of 200 nm by thermoplastic nanomolding at 25kN, which we have described in detail elsewhere^17^. Surface roughness was characterized using AFM images. We found the surface roughness of flat BMG equaled 14.1 ± 2.8 nm, nanopatterned BMG equaled 231.7 ± 47 nm, and titanium equaled 55.6 ± 8.1 nm. In addition, we analyzed surface chemistry using x-ray photoelectron spectroscopy (XPS), which showed expected elemental compositional components for both Pt-BMG and Ti based alloys (Fig. 1c). Furthermore, the XPS survey showed similar peaks and spectra for flat and nanopatterned BMG indicating that nanopatterning did not change elemental surface composition. We also compared the reported mechanical properties for titanium^41^ and Pt-BMG^8^ and show that the lower elastic modulus of the latter does not diminish yield and tensile strength (Fig. 1d). The effect of nanostructuring on material properties is quite small. While ductility may increase slightly when confined to the nanoscale^42^, modulus is unchanged^43^.

### MSC viability and morphology on substrates

Tissue culture polystyrene (C_8_H_8_) was chosen as a control substrate because of its application in most *in vitro* studies of cell differentiation. We evaluated cell viability by culturing hMSCs on nanopatterned Pt-BMG, titanium, and TCP for 24 hours. To confirm biocompatibility, a live/dead assay was performed; results indicate that hMSCs have a 90 percent or higher viability on all three substrates, with the highest percentage (97.8) on flat Pt-BMG. No significant differences were seen in viability across all substrates (Figure S1).

**Figure 2:**
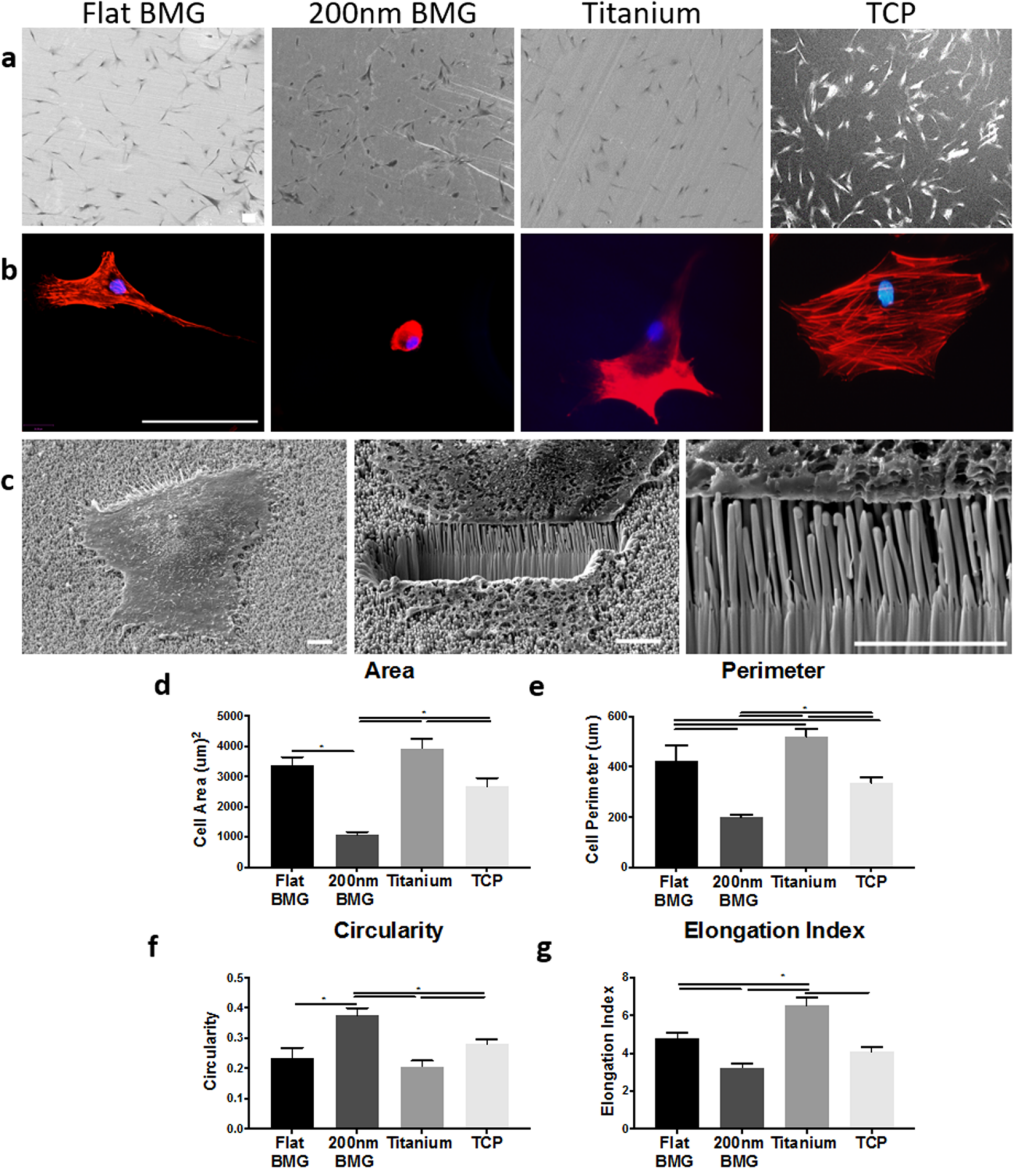
Mesenchymal stem cell morphology on substrates. (**a**) SEM of hMSCs on flat Pt-BMG, 200 nm Pt-BMG, titanium, and tissue culture plastic, scale bar = 100 μm. (**b**) Representative images of hMSCs stained with phalloidin (cytoskeleton, red) and DAPI (nucleus, blue), scale bar = 50 μm. (**c**) FIB-SEM images of a hMSCs on 200 nm Pt-BMG, scale bar = 5 μm. (**d–g**) Quantification of cell area, perimeter, circularity, and elongation index (one-way ANOVA with post hoc Tukey HSD test, n = 50 cells, *p < 0.05). Error bars represent standard error of the mean (SEM).

The impact of substrate chemistry and topography on hMSC morphology was then characterized at 24 hours. Qualitative assessment of morphology was performed using SEM, which showed cell attachment and spreading on all surfaces. However, hMSC spreading was reduced on 200 nm Pt-BMG in comparison to the flat Pt-BMG, titanium, and TCP. Cells on 200 nm Pt-BMG appeared smaller and more circular, while cells were larger and more spread on the other substrates (Figs 2a,b, S2). Additionally, focused ion beam scanning electron microscopy (FIB-SEM) was used to analyze hMSC interaction with the nanorods. Cells are shown to be suspended on the nanorods indicating that they interact directly with the top of these nanotopographical features (Fig. 2c). Image J analysis of cells stained with rhodamine-phalloidin to visualize F-actin confirmed these observations. Cell area and perimeter were greater on flat Pt-BMG and titanium in comparison to nanopatterned Pt-BMG, indicating that nanotopography affected hMSC morphology (Fig. 2d,e). Interestingly, changes were observed in cell perimeter and circularity between flat Pt-BMG and titanium, suggesting that substrate material composition alone influenced cell spreading. Circularity was also reduced and elongation index was higher for cells on flat Pt-BMG and titanium, in comparison to nanopatterned Pt-BMG (Fig. 2f,g). MSC adhesion was also assayed. Briefly. cells were cultured at the same density used in the differentiation studies. Nuclei was counted after 24 hours and no difference was observed across substrates suggesting that adhesion was not compromised (Figure S3). Overall, these results indicate that nanotopography and substrate composition modulate hMSC size and spreading.

### Osteogenic and adipogenic differentiation of hMSCs on substrates

**Figure 3:**
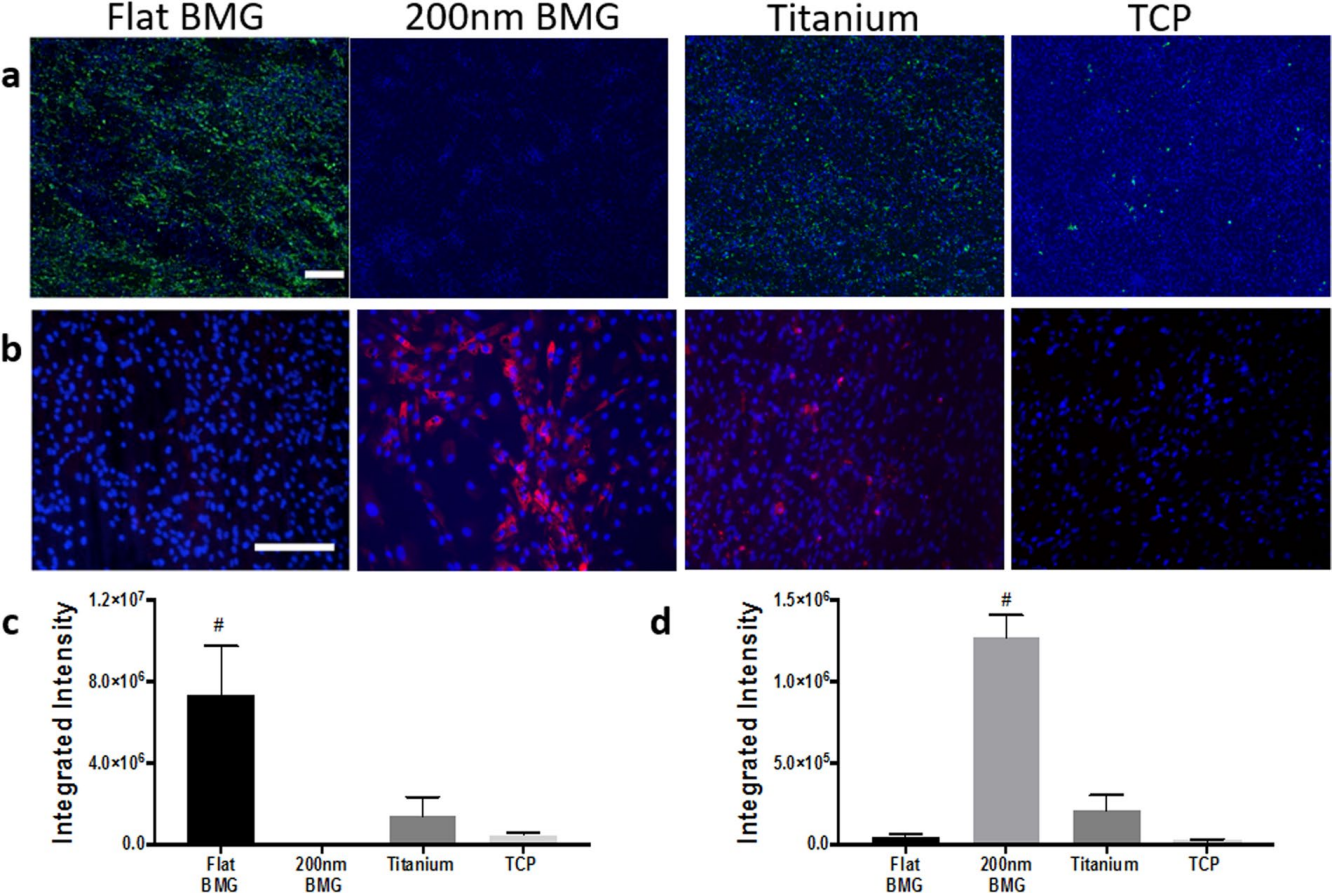
Differentiation on substrates. (**a**) Mineralization fluorescence on all substrates (in green), scale bar = 200 μm (**b**) Adiponectin fluorescence on all substrates (in red), scale bar = 100 μm (**c**) Quantification of mineralization fluorescence (one-way ANOVA with post hoc Tukey HSD test, n ≥ 3 samples, at least 5 images per sample, ^#^p < 0.05 relative to flat BMG) (**d**) Quantification of adiponectin fluorescence (one-way ANOVA with post hoc Tukey HSD test, n = 3 samples, at least 15 images per sample, ^#^p < 0.05 relative to 200 nm BMG). DAPI was used for nuclear staining (in blue). Error bars represent standard error of the mean (SEM).

To evaluate the biomedical potential of these materials, we assayed adipogenic and osteogenic differentiation by culturing hMSCs on each substrate in mixed media for 14 days. Mixed media was constructed with equal amounts of adipogenic and osteogenic factors. We analyzed the substrates for mineralization to determine the extent of differentiation into osteoblasts, as evidenced by hydroxyapatite deposited as bone-like nodules. As shown in Fig. 3a, flat Pt-BMG substrate induced the highest level of mineralization; thus, cells resident on this substrate were more likely to become osteogenic cells. In contrast, little mineralization was observed on the 200 nm Pt-BMG. Interestingly, flat Pt-BMG encouraged more osteogenic differentiation than titanium, which is the gold standard for orthopaedic applications. We also observed that osteogenesis was impaired on the 200 nm Pt-BMG (Fig. 3a,c). Adiponectin, a protein secreted by adipose tissue, was detected in order to assess adipogenesis. As shown in Fig. 3d, the efficacy of adipogenic differentiation was higher on the 200 nm Pt-BMG than on both flat Pt-BMG and titanium. Furthermore, adipogenesis was higher on titanium compared to flat Pt-BMG (Fig. 3b,d). We also assessed differentiation on flat and nanopatterned BMG without the addition of differentiation supplements and see no significant difference in adiponectin fluorescence between these groups (Figure S4). We also observed an 81-fold increase in adiponectin fluorescence on 200 nm Pt-BMG and a 17-fold increase in mineralization on flat Pt-BMG in comparison to TCP. Overall, these data demonstrate that topography and material composition in conjunction with differentiation factors have a profound influence on stem cell differentiation.

### Mechanisms of differentiation

**Figure 4:**
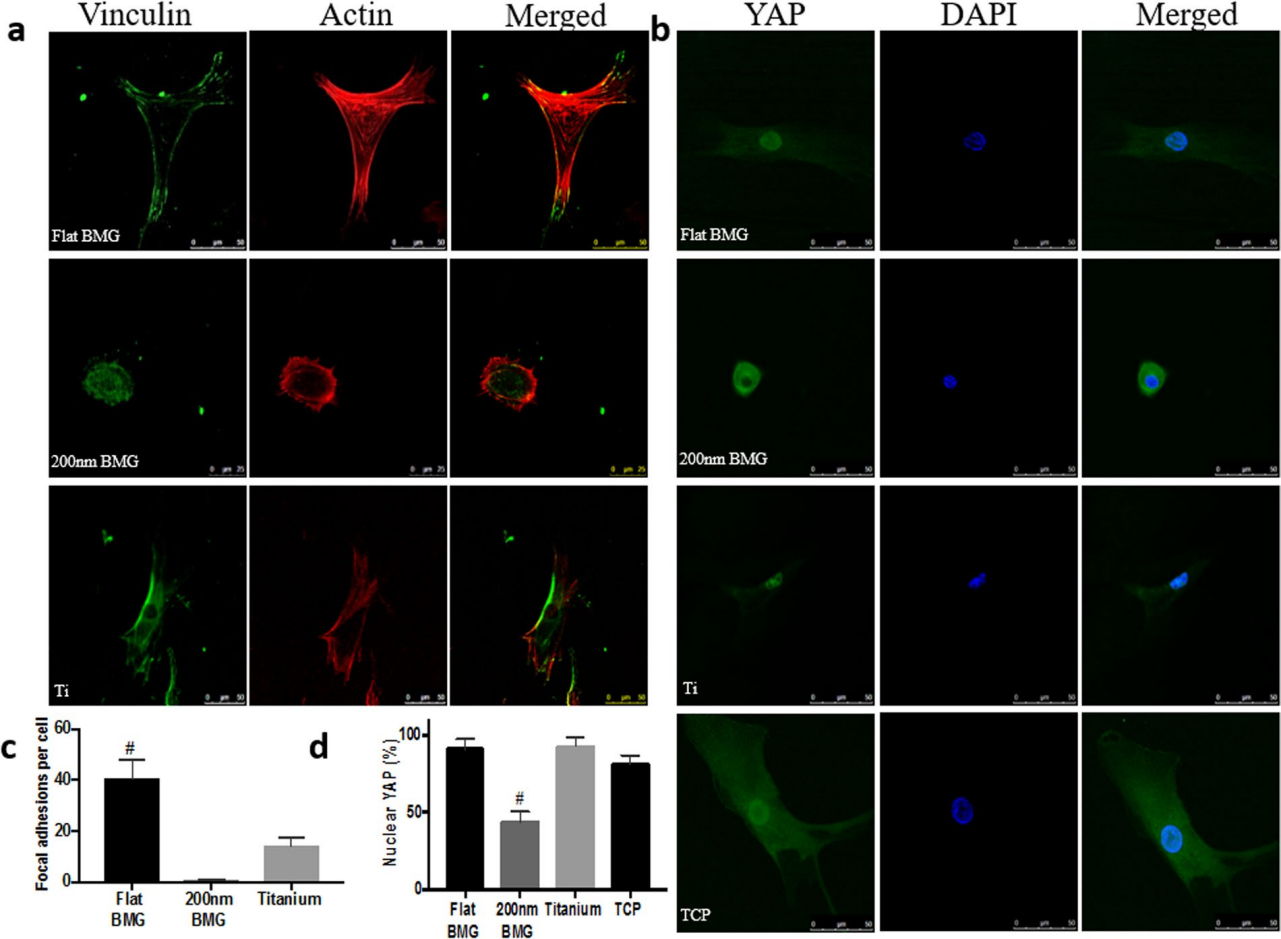
Mechanotransduction on substrates. (**a**) Confocal micrographs for vinculin, a focal adhesion protein (in green). (**b**) Immunofluorescence of YAP (in green). (**c**) Quantification of focal adhesion per cell (one-way ANOVA with post hoc Tukey HSD test, n = 3 samples, at least 8 cells per sample, ^#^p < 0.05 in comparison to flat BMG). (**d**) Quantification of nuclear YAP (one-way ANOVA with post hoc Tukey HSD test, n = 3 samples, at least 40 cells per sample, ^#^p < 0.05 in comparison to 200 nm BMG). Error bars represent standard error of the mean (SEM).

Mechanical signals induced by surface chemistry and topography are converted into biochemical signals to regulate cell behavior^44^. This can occur through biomolecular changes or propagation of mechanical forces via the cytoskeleton into the nucleus. Based on the differences observed in differentiation efficacy, signaling mechanisms that regulate how substrate-induced signals are transduced to regulate gene expression were studied. Specifically, focal adhesion formation and the activation of specific genetic programs (YAP) were investigated. Focal adhesions play an essential role in sensing ECM stiffness and transducing information to integrins that regulate cell differentiation. Adherent cells sense and respond to physical cues by actively modifying focal adhesion assembly and signaling^45^. Cells were plated on each substrate for 24 hours and then stained for vinculin or YAP to investigate early signaling events. Previous studies have demonstrated that osteogenic differentiation increases with a higher number of focal adhesions via regulation of RUNX2, a transcriptional regulator of osteogenesis^46^. Cells stained for vinculin showed more focal adhesions on flat Pt-BMG than on titanium or 200 nm Pt-BMG (Fig. 4a,c).

To investigate the activation of genetic programs, we assayed subcellular localization of YAP. YAP, a nuclear transducers of the Hippo pathway, has been shown to change localization on stiff and micropatterned extracellular matrix (ECM) to regulate differentiation of MSCs in adipogenic and osteogenic lineages^33^. Specifically, nuclear localization corresponds to osteogenesis, while a loss of nuclear YAP corresponds to adipogenesis. Cells stained for YAP showed higher nuclear localization on the flat Pt-BMG, titanium, and TCP compared to the 200 nm Pt-BMG (Fig. 4b,d). Interestingly, results indicated that titanium induced an intermediate level of osteogenesis. Although similar YAP localization is seen as on flat Pt-BMG, the reduction of focal adhesions of hMSCs on titanium may prevent efficient transcription of osteogenic inputs.

### Mineralization of hMSCs with biomechanical and chemical modifications

**Figure 5:**
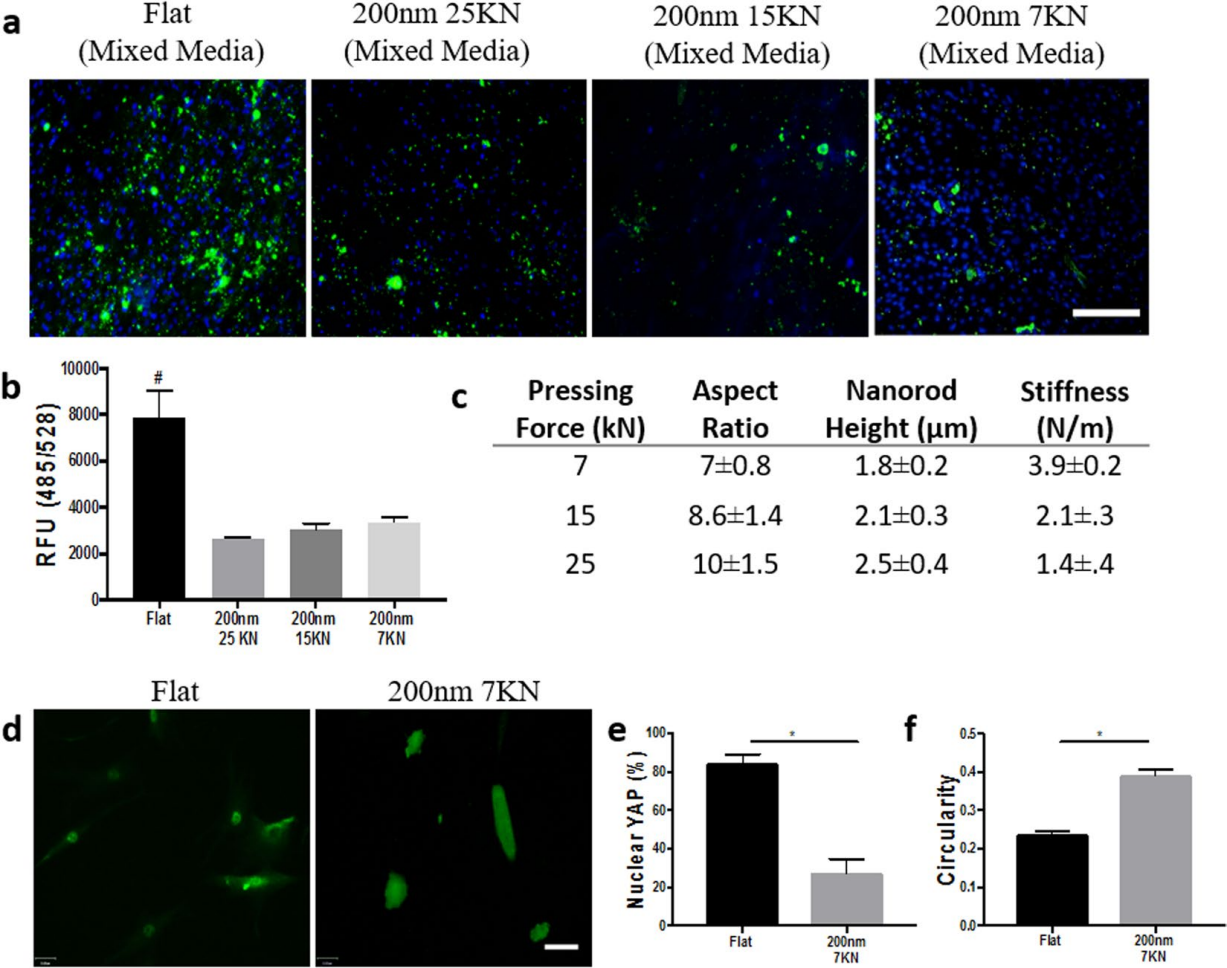
Mineralization with experimental modifications. (**a**) Mineralization fluorescence for aspect ratio modifications of 200 nm Pt-BMG (in green), DAPI was used for nuclear staining (in blue). scale bar = 100 μm (**b**) Quantification of mineralization fluorescence on modifications (one-way ANOVA with post hoc Tukey HSD test, n = 3 samples, ^#^p < 0.05 in comparison to flat BMG). (**c**) Theoretical stiffness values. (**d**) YAP fluorescence of hMSCs cultured on flat Pt-BMG and 200 nm BMG pressed at 7KN (in green), scale bar = 100 μm (**e**) Calculation of YAP nuclear localization fluorescence (student’s t-test, n = 3 samples, at least 40 cells per sample *p < 0.05). (**f**) Quantification of hMSC circularity (student’s t-test, n > 40 cells, *p < 0.05). Error bars represent standard error of the mean (SEM).

To further investigate the effect of biomechanical modifications on MSC differentiation, we adjusted the stiffness of individual 200 nm nanorods by reducing forming pressure to control filling depth during thermoplastic forming in order to manipulate aspect ratio. Lower forming pressure results in shorter and stiffer rods^17^. Numerous studies have indicated that cells are able to sense material stiffness and respond in a manner similar to the native tissue in which they reside^47^ These reports have mostly focused on polymers of different stiffness which when taken together, suggest that stiffer substrates are more likely to produce osteogenic cells compared to less stiff substrates. To determine if mineralization is increased on stiffer substrates, we plated cells on 200 nm nanorods formed at 7 kN, 15 kN and 25 kN with stiffnesses of 3.9, 2.1, and 1.4 N/m, respectively. Cells were assayed for mineralization at 14 days, and no differences were observed between the nanopatterned Pt-BMG substrates. Suggesting that osteogenic differentiation on Pt-BMGs, within the range examined, is mediated primarily by topography (Fig. 5a–c).

In earlier experiments, we discovered that YAP localization is less nuclear on taller BMG nanorods. To understand the mechanism driving osteogenic differentiation on shorter and stiffer nanotopographical surfaces, we assayed YAP signaling. HMSCs were cultured on BMGs with 200 nm nanorods pressed at 7KN. The lack of nuclear YAP localization is preserved on these stiffer nanorods as compared to flat BMG (Fig. 5d,e). Furthermore, nuclear localization is similar for 200 nm BMGs pressed at 7KN and 25KN. It has been suggested that the rounded cells display inhibition of YAP^48^. To assay if cell roundedness is maintained on stiffer rods, we cultured hMSCs on nanopatterned BMGs pressed at 7KN and stained for F-actin. Utilizing flat BMGs as a control, hMSCs were found to be more circular (Fig. 5f) and circularity was similar on stiffer rods pressed at 7KN and less stiff rods pressed at 25KN. Overall, changing the stiffness and the aspect ratio of the rods did not affect differentiation because cell roundedness is preserved.

**Figure 6:**
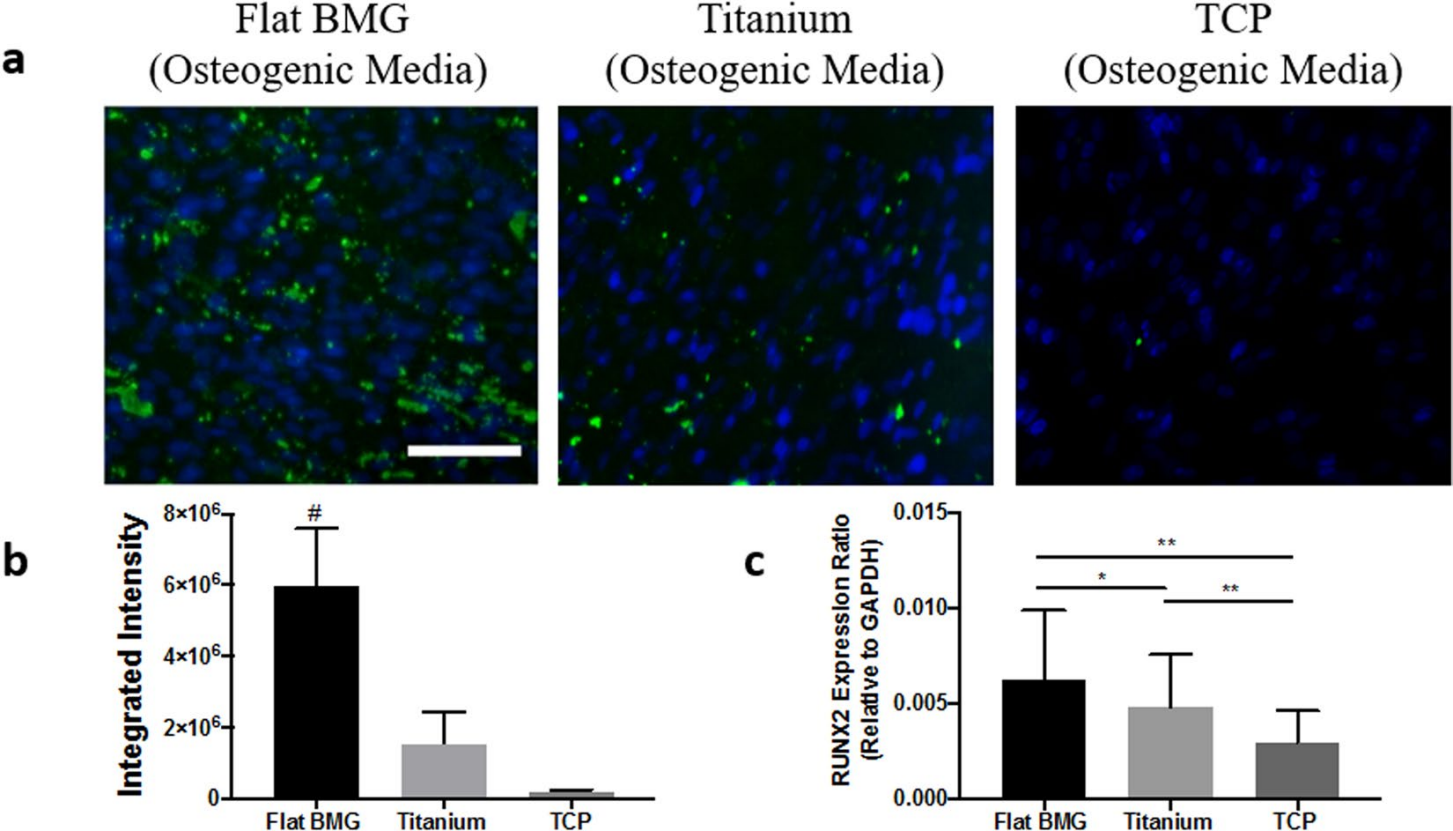
Mineralization in osteogenic media alone. (**a**) Mineralization fluorescence of hMSCs cultured in osteogenic media on flat Pt-BMG and titanium (in green), scale bar = 100 μm (**b**) Calculation of mineralization fluorescence (one-way ANOVA with post hoc Bonferroni-Holm test, n = 3 samples, at least 5 images per sample, ^#^p < 0.05 in comparison to flat BMG). (**c**) PCR for RUNX2, an osteogenic marker (one-way ANOVA with post hoc Tukey HSD test, n = 3 samples, *p < 0.05, **p < 0.01). Error bars represent standard error of the mean (SEM).

Finally, to assess the effect of osteogenic media alone on differentiation of cells on titanium and flat Pt-BMG, we cultured hMSCs on these substrates for 14 days in osteogenic media and then assayed mineralization. Mineralization was also higher on flat Pt-BMG in comparison to titanium and TCP, in a similar manner to what was observed in mixed media (Fig. 6a,b). Consistent with this observation, RUNX2 expression was found to be greatest on flat BMG (Fig. 6c).

## Conclusion

MSC differentiation is mediated by complex material properties including topography composition, and biomechanics. In the present study, we observed that nanotopography overrides biochemical cues in that MSCs preferentially differentiate into osteoblasts on flat Pt-BMG and adipocytes on 200 nm Pt-BMG, regardless of similar elemental composition and biochemical inputs. Previously, studies have shown that nanotopography can control cell adhesion, differentiation, affect mechanical properties and produce specific biological responses. It is appreciated that nanoscale feature sizes are similar to that of cell adhesion receptors and allow for better control of signaling pathways mediated by integrins^35^. While reports on the effect of nanotopography on the osteogenic differentiation of stem cells at various moduli abound^49,50^, there is a paucity of studies examining the nanotopographical effects on adipogenesis for materials over 80 GPa^51^. Here we show that the latter is mediated by a reduction in focal adhesions and nuclear YAP, which together attenuate transcription of osteogenic pathways while activating adipogenic pathways. On 200 nm Pt-BMGs, nanotopography confines the degree of hMSC spreading leading to the inactivation of YAP that occurs through its localization to the cytoplasm to promote adipogenesis^48^. Nanopatterned Pt-BMGs display homogenous features. The observed cell responses suggest that topography has a critical and oft-ignored role in regulating adipogenic differentiation. Moreover, 200 nm nanotopography on a high elastic moduli material improves adipogenic differentiation of stem cells. Interestingly, a previous study proposed 15 nm hollow tubes to be ideal for osteogenesis on metallic implants^52^. Therefore it is possible that smaller scale nanotopography increases osteogenesis whereas larger scale nanotopography improves adipogenesis. It is critical to note while some studies use nanotopographical features on titanium to increase osteogenic differentiation^52,53^, we see a reduction in osteogenesis on Pt-BMG nanopatterns indicating the complex interaction of material composition and topography in controlling stem cell lineage. Although comparison of nanostructured BMG to nanostructured titanium is of interest, the crystalline nature of titanium prevents its thermoplastic forming and direct comparison.

Previously, it has been shown that vertical quartz nanopillars induce cytoskeletal-dependent nuclear deformation in fibroblasts^54^. Importantly, fibroblasts were shown to interact directly with the nanopillars, the space between nanopillars, and the substrate. A separate study investigated nuclear deformation of fibroblasts cultured on PDMS nanogratings or nanopillars^55^. On the latter, they described a reduction in the size of focal adhesions when cells were exposed to nanopillars with diameters of 500 or 1,000 nm. Fabrication of smaller nanopillars, in the 300 nm range, was not feasible due to clamping. Here, we show that the interaction of MSCs with BMG-200 is restricted to the top surface of the nanorods, as shown by FIB-SEM. Therefore, we conclude that in our system, MSCs are exposed to a reduced surface area leading to reduced cell size, rounded morphology and suboptimal focal adhesion formation.

Current approaches to understanding MSC differentiation *in vitro* involve low elastic moduli substrates such as hydrogels and polymers that are suboptimal for load-bearing applications^56,57^. In an effort to engineer biological responses, studies have described surface modifications and functionalization to alter material mechanical properties in order to control cell adhesion and differentiation^57,58^. Moreover, studies that analyzed the impact of matrix stiffness on differentiation show that stem cells differentiate on substrates closest to the stiffness of their native environment, suggesting that adipogenesis should be improved on substrates with moduli similar to adipose tissue (10^3^–10^4^ Pa)^28,59–61^. Surprisingly, we observed significant adipogenesis on stiff substrates (95 GPa) with topographical features. Furthermore, when cells are differentiated on nanorods with increasing stiffness, we did not observe a shift to osteogenic levels, indicating that nanorod diameter has a greater influence than nanorod stiffness on directing differentiation.

Analyses of osteogenic signaling pathways show similar YAP localization on flat BMG and titanium. A reduction of focal adhesion formation on titanium could prevent efficient transcription of RUNX2 and suppress differentiation into bone-like cells. This behavior could potentially be mediated by the composition of these metals. Studies have shown that the extracellular domains of integrins preferentially bind to divalent cations exposed on the surface of a biomaterial and activate signaling pathways that mediate cell proliferation and differentiation and stabilize osteogenic phenotypes^59^. As a result, we hypothesize that the presence of divalent ions, nickel and copper, lead to preferential osteogenesis on Pt-BMG in comparison to titanium. Furthermore, the lower surface roughness of the flat BMG in comparison to titanium may influence differentiation and signaling by changing protein adsorption. Taken together, these results highlight the potential use of Pt-BMGs for orthopaedic applications.

In this study, efficient differentiation was achieved without the addition of extensive chemical and structural modifications. Pt-BMGs functioned as regulators of stem cell fate with flat Pt-BMGs as a substrate for *in vitro* osteogenesis. Furthermore, the limitations of titanium are overcome without compromising the need for high strength materials in load-bearing applications. Surface nanopatterning enables cells to overlook biophysical cues from a high moduli material to form adipocytes. By confining cell shape using nanotopography, we can control specific stem cell responses. Moreover, Pt-BMGs could also be utilized in systems where *in vitro* differentiation is inefficient, such as neuronal^62^ and myogenic^63^ differentiation of MSCs since the amorphous nature of Pt-BMGs enables facile, reproducible, and tunable creation of surface modifications. Moving forward, this work adds credence to the use of Pt-BMGs as bioactive and functional replacement of titanium components for better clinical outcomes.

## Materials and Methods

### Substrate Fabrication

An amorphous, bulk platinum alloy was cast using high purity materials made of platinum, copper, nickel, and phosphorus, as described previously^10^. The bulk alloy was saw diced into sections and fabricated using thermoplastic forming. Briefly, for flat samples, cut Pt-BMG was heated at 270 °C and pressed using an Instron. Alumina Oxide (AAO) inorganic filter membranes with 200 nm pores (Whatman) were purchased and used as templates for nanopatterned samples. To fabricate 200 nm samples, Pt-BMG was heated with a membrane at 270 °C at a force of 7–25KN. Material stiffness was calculated as stated previously^16^. Briefly, nanorod length were calculated using ImageJ. Nanorod stiffness was calculated as k = (3πED^4^)/64L^3^. Where k represents nominal nanorod stiffness, E represents elastic modulus, D represents nanorod diameter and L represents nanorod length. Nanopatterned Pt-BMG was etched in a 30% potassium hydroxide (KOH) solution. Samples were inspected by SEM to ensure complete alumina dissolution. Grade 5 titanium (McMaster-Carr) and polystyrene (Falcon) were commercially purchased. All samples were cleaned with acetone, isopropanol, ethanol and PBS washes prior to cell studies.

### Substrate Characterization

For SEM, all substrates were visualized with a Hitachi SU-70 SEM. For cell studies, hMSCs were incubated on substrates in maintenance media for 24 hours. Substrates were washed in PBS and incubated in 1% glutaraldehyde in for 10 minutes, washed in distilled water for 5 minutes and dehydrated in 75, 80, 95, and 100% ethanol, then incubated in hexamethylididilazane (HMDS) for 10 minutes and air dried. Samples were sputter coated with chromium or iridium and imaged. A dual FIB-SEM was used to mill cross sections of hMSCs on 200 nm nanorods and to obtain images.

For AFM, samples were cleaned in ethanol and imaged using a Bruker Dimension Icon AFM. Roughness was calculated using 10 um × 10 um images. Images were measured by Bruker Multimode AFM with Nanoscope III electronics and Bruker RTESPAW-300 silicon cantilevers with driving frequencies ranging from 290 kHz to 300 kHz. RMS roughness was calculated directly by the software (WxSM). Three AFM images were analyzed with an n number of 3 samples. Surface chemistry via XPS was acquired using a PHI VersaProbe II XPS microprobe.

### *In Vitro* Cell Culture and Differentiation

Adipose derived hMSCs were maintained in low glucose DMEM containing 10% fetal bovine serum and 1% penicillin-streptomycin at 37 °C and 5% CO_2_. Cells were maintained and expanded on plasma treated tissue culture dishes with media changes every three days. Passage 2–7 cells were used for all experiments. At 80–90% confluence, cells were detached with trypsin and plated at 30,000 cells/well on substrates in 24 well plates. For differentiation in mixed media, cells were incubated in StemXVivo Osteogenic/Adipogenic Base Media supplemented with human StemXVivo osteogenic supplement and StemXVivo adipogenic supplement (R&D systems) for 14 days. For differentiation in osteogenic media, cells were incubated in culture media supplemented with human StemXVivo osteogenic supplement for 14 days. For no induction control, cells were incubated in maintenance media.

### Quantification of Cell Morphology

Cells were plated at a low density in maintenance media for 24 hours. Cells were fixed with 4% PFA and stained with Rhodamine Phalloidin (Invitrogen) and DAPI. Individual cells were manually outlined using ImageJ. Area, perimeter, circularity and elongation index were quantified for at least 50 cells per substrate. Circularity was defined as [(4π(cell area)/(cell perimeter)^2^]. Elongation index was defined as [(cell perimeter)^2^/(4π(cell area))].

### Mineralization Assay

Mineralization was assayed using an OsteoImage Bone Mineralization Assay (Lonza) based on the binding of a fluorescent reagent to hydroxyapatite on bone-like nodules deposited by osteogenic cells. Media was aspirated, and cells were washed in PBS. Cells were fixed with 4% PFA, washed, and incubated in the OsteoImage reagent for 30 minutes. For quantitative analysis, wash buffer was added to each well and analyzed using a fluorescence plate reader at excitation/emission wavelengths (485 nm/525 nm). Substrates were mounted on glass slides for fluorescence microscopy (Zeiss). At least five images (2.5×) per sample (n = 3) were quantified using MetaMorph software (Molecular Devices) to measure relative integrated intensity per image of the fluorescent stains.

### Immunofluorescence

Cells were cultured on disks at specific time points. Media was aspirated, and disks were washed in PBS. Cells were fixed with 4% PFA and permeabilized, incubated in blocking buffer, and then antibodies against adiponectin (Abcam, ab22554), vinculin (Abcam, ab18058), or YAP (SCBT, sc101199) overnight. Disks were washed, incubated in the appropriate second antibody for one hour, and mounted on glass slides for microcopy using a fluorescent microscope. Immunofluorescence images for adipogenesis and osteogenesis were quantified using MetaMorph. Vinculin and YAP were imaged with a fluorescent microscope and confocal microscope (Leica). YAP was manually quantified as predominantly nuclear versus cytoplasmic^33^.

### Quantitative Real Time PCR

RNA was isolated with an RNeasy Mini Kit (Qiagen) and reverse transcribed with a QuantiTect Reverse Transcription Kit (Qiagen). Reverse transcriptase polymerase chain reactions (RT-PCR) was performed in triplicate using primers for RUNX2 with GAPDH as a control. Relative expression ratios are reported. RUNX2 *forward* (5′-CCAGATGGGACTGTGGTTACC-3′), *reverse (*5′-ACTTGGTGCAGAGTTCAGGG-3′). GAPDH *forward* (5′-AAGTGGATATTGTTGCCATC-3′), *reverse (*5′-ACTGTGGTCATGAGTCCTTC-3′*)*.

### Statistical Analysis

Error bars represent standard error of the mean (SEM). Data is presented as mean ± SEM. An unpaired student’s t-test was used when comparing two groups. For comparisons between three or four groups, one-way analysis of variance (ANOVA) tests with post hoc tests were utilized as stated.

## Electronic supplementary material

Supplemental Information
