# Supplementary material for: Regulation of Mesenchymal Stem Cell Differentiation by Nanopatterning of Bulk Metallic Glass
Source: Sci Rep. 2018 Jun 8;8:8758. doi: 10.1038/s41598-018-27098-6 (PMC5993820; doi:10.1038/s41598-018-27098-6)
Supplement: Supplementary file 1 — Supplemental Information [file 41598_2018_27098_MOESM1_ESM.docx]

**Supplementary Information**

**Regulation of Mesenchymal Stem Cell Differentiation by Nanopatterning of Bulk Metallic Glass**

*Ayomiposi M. Loye^a,b^, Emily R. Kinser^a,c,d^, Sabrine Bensouda^a^, Mahdis Shayan^a,b^, Rose Davis^e^, Rui Wang^d^, Zheng Chen ^a,c^, Udo D. Schwarz ^a,c,f^*, *Jan Schroers^a,c^, Themis R. Kyriakides^a,b.g*^*

**a. Center for Research on Interface Structures and Phenomena, Yale University, New Haven,**

**CT 06520, USA**

**b. Department of Biomedical Engineering, Yale University, New Haven, CT 06520, USA**

**c. Department of Mechanical Engineering and Materials Science, Yale University, New Haven, CT 06520, USA**

**d.** **IBM Thomas J. Watson Research Center, New York, NY 10598, USA**

**e. Department of Molecular, Cellular and Developmental Biology, Yale University, New**

**Haven, CT 06520, USA**

**f. Department of Chemical and Enviromental Engineering, Yale University, P.O. Box 208089,**

**New Haven, CT 06520, USA**

**g. Department of Pathology, Yale University, P.O. Box 208089, New Haven, CT 06520, USA**

* correspondence to themis.kyriakides@yale.edu


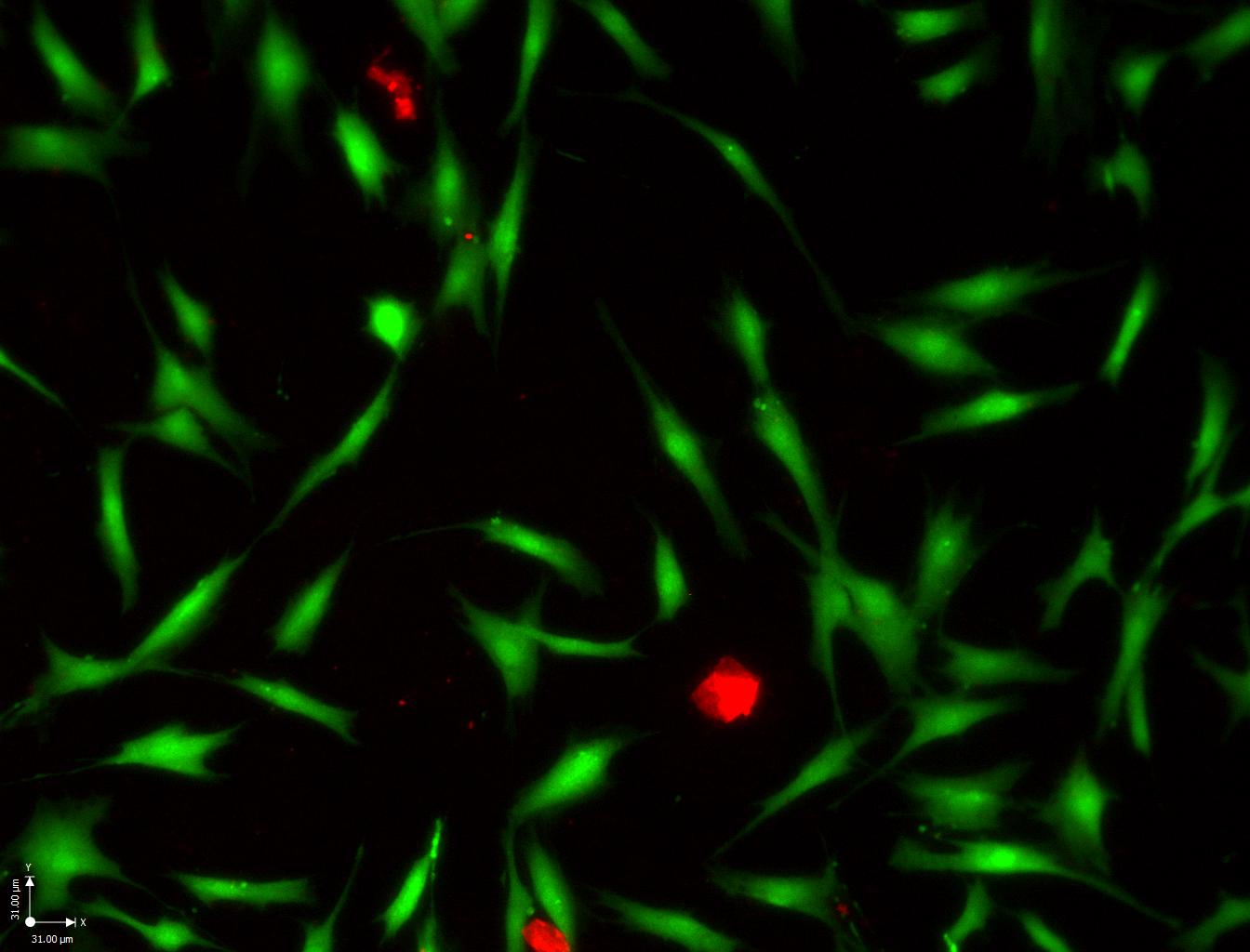

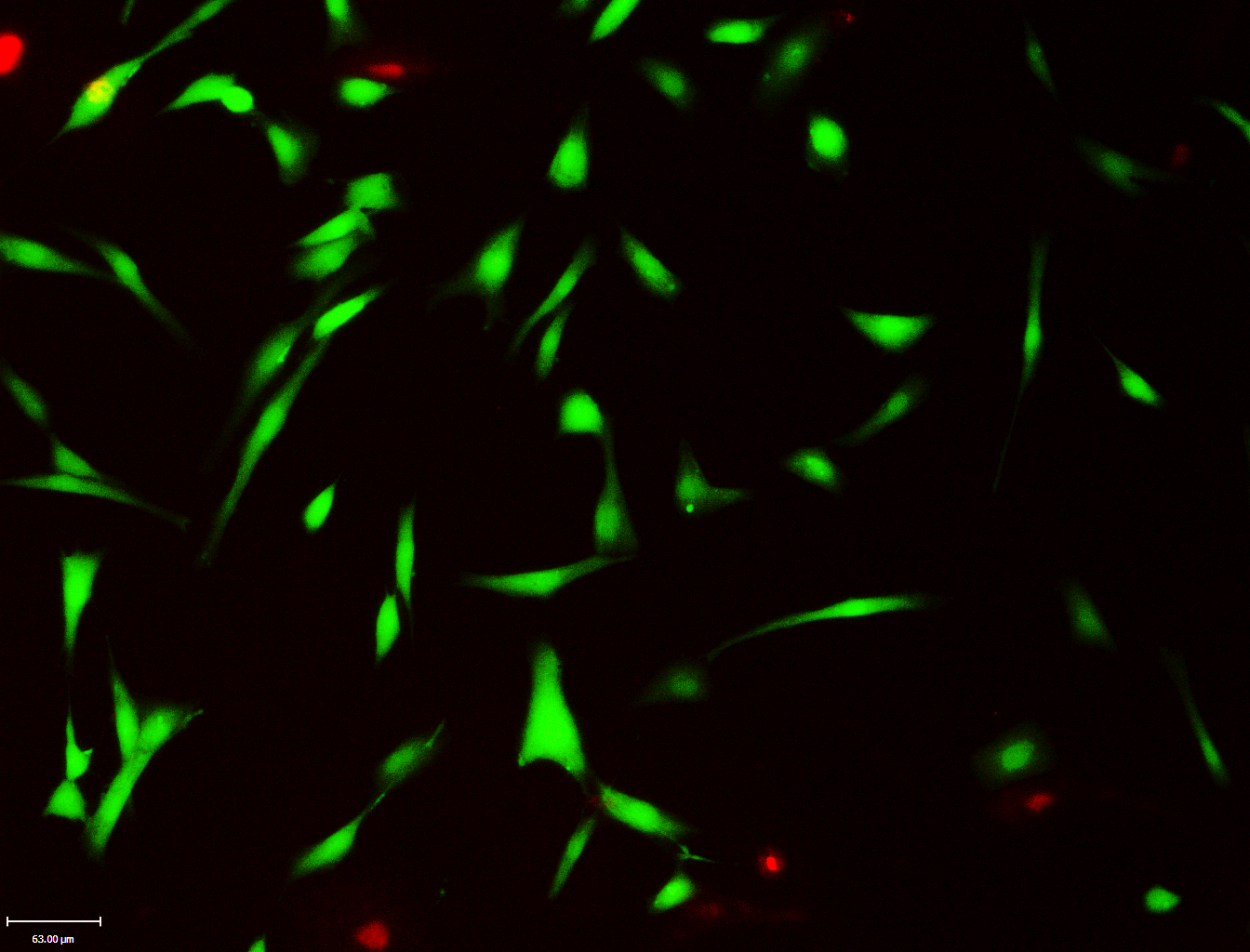

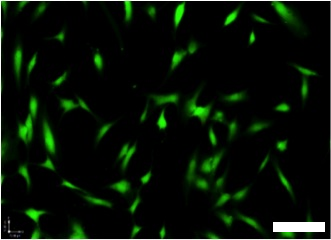


**a**

**b**

**c**

d

**Supplemental Figure 1.** Live/dead assay of cells on (a) flat BMG, (b) 200nm BMG, and (c) titanium. (d) Quantification of live/dead assay. Viability was assayed with a live/dead assay (Invitrogen). Cells were cultured on each substrate for 48 hours, washed, stained with calcein-AM (live-green) and ethidium homodimer-1 (dead-red) for 10 minutes, and imaged. (n=3 samples, at least 200 cells per sample, one-way ANOVA with post hoc Tukey HSD test, ns, scale bar = 100 μm). Error bars represent standard error mean (SEM).


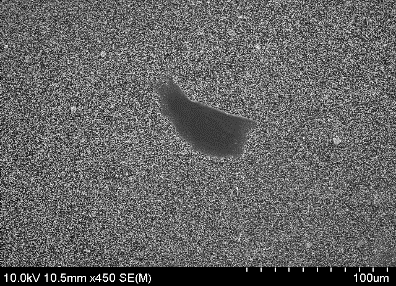

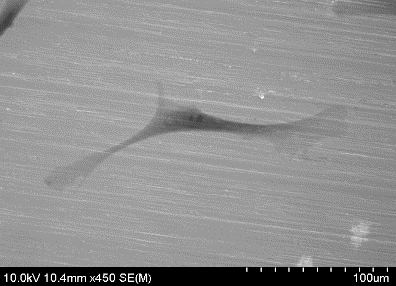

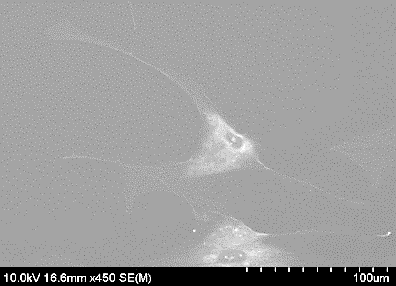

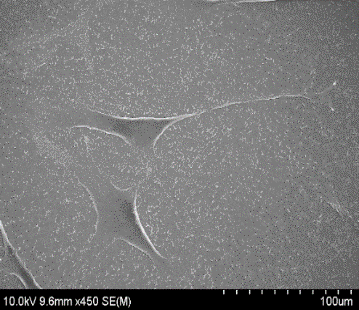


**Supplemental Figure 2.** Higher magnification SEM of hMSCs on (a) flat BMG, (b) 200nm BMG, (c) titanium, and (d) tissue culture plastic.

**a**

**b**

c

d


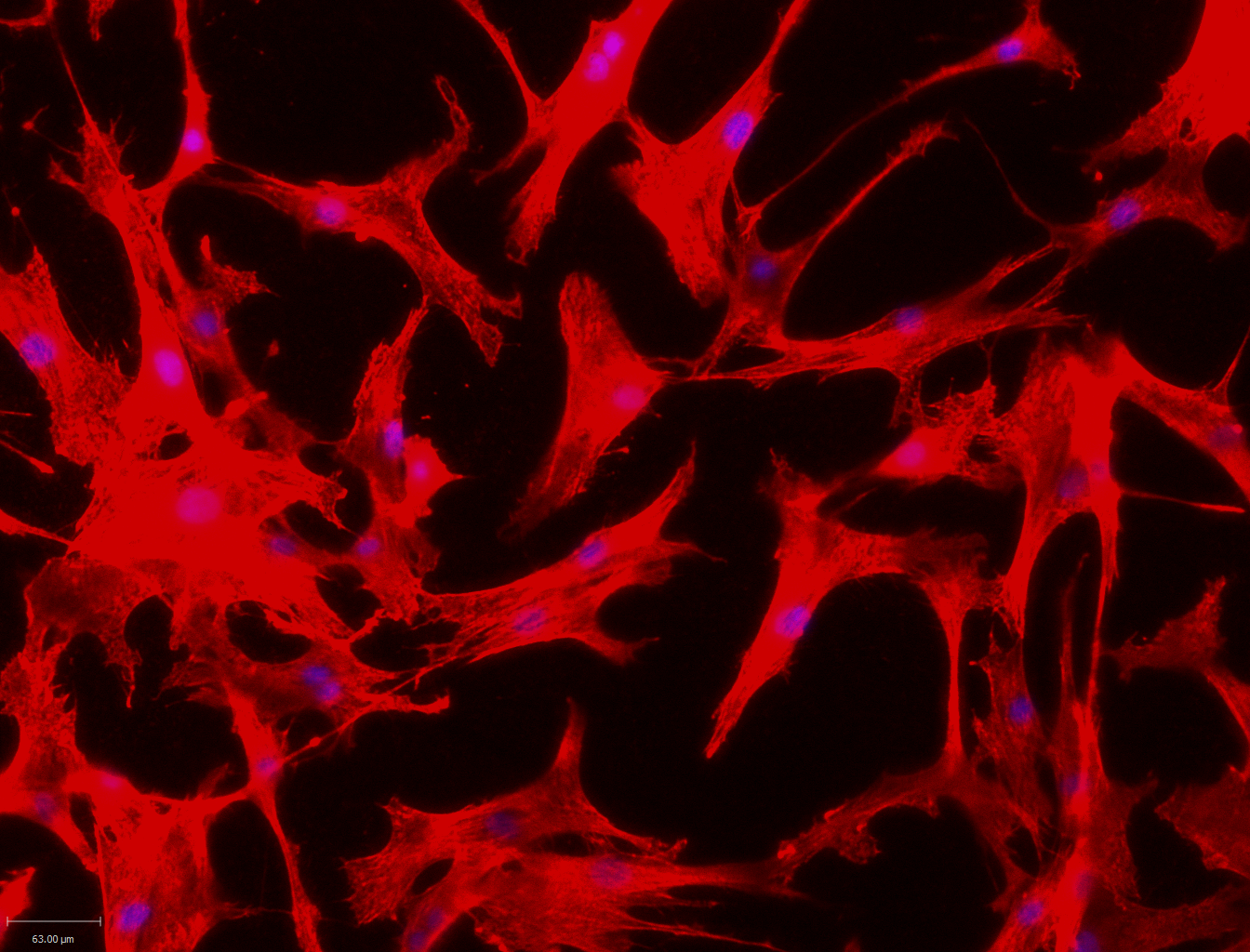

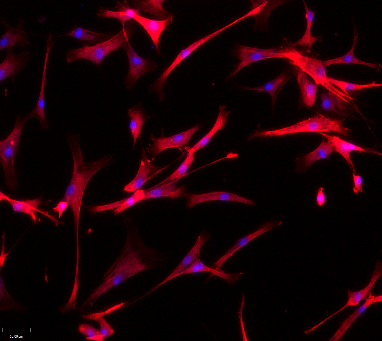

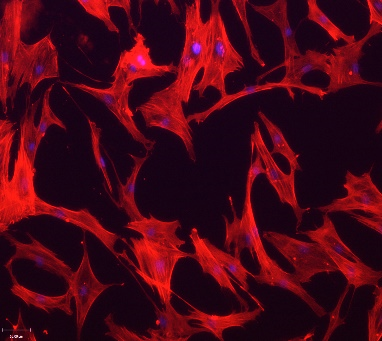


c

b

d

e

**Supplemental Figure 3.** MSC adhesion on (a) flat BMG, (b) 200nm BMG, (c) titanium, and (d) tissue culture plastic. (e) Quantification of adhesion. Cells were cultured on each substrate for 24 hours and stained with rhodamine-phalloidin. Nuclei was manually counted (n=3 samples, at least 200 cells per sample, one-way ANOVA with post hoc Tukey HSD test, not significant, scale bar = 100 μm). Error bars represent standard error mean (SEM).


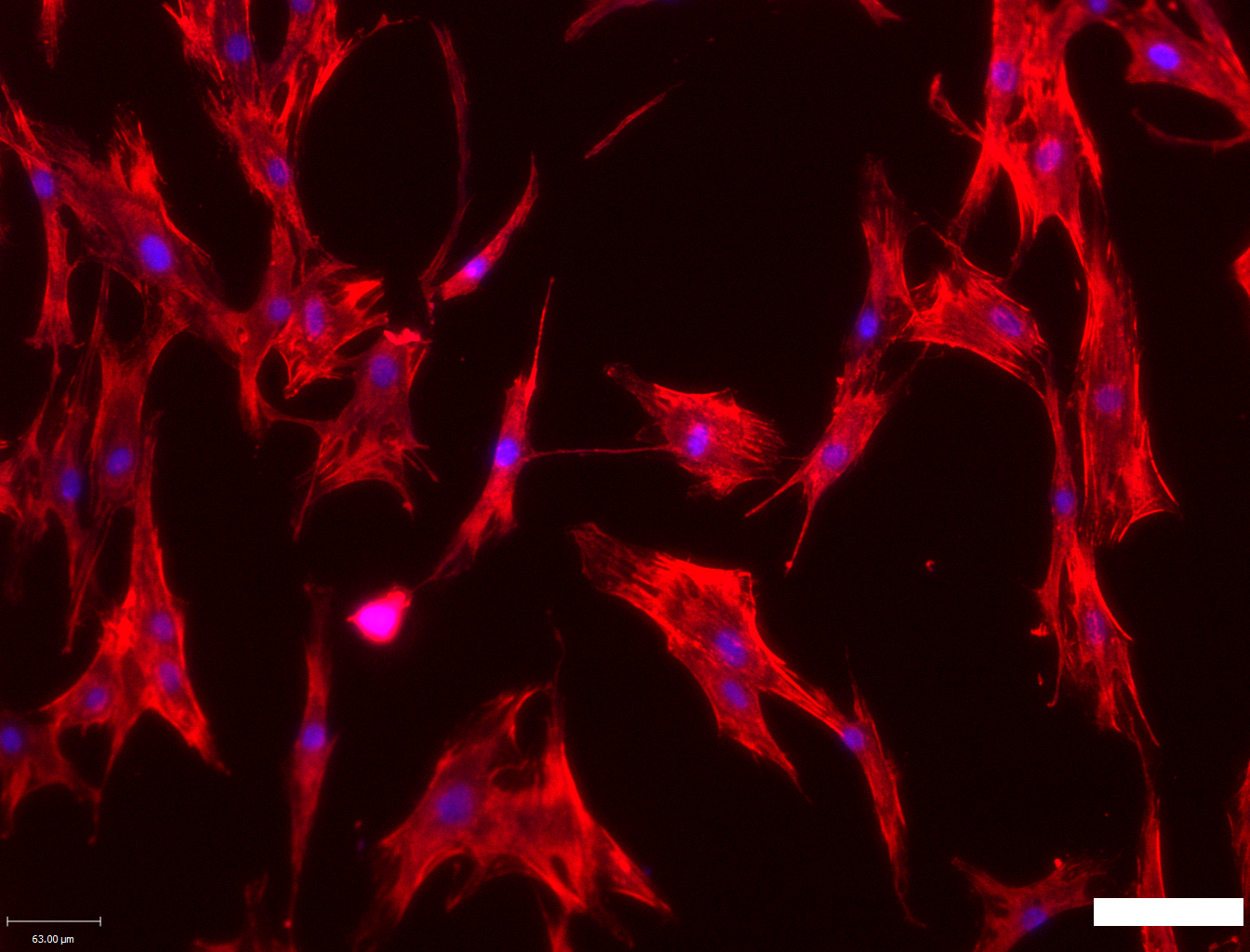


a

**Supplemental Figure 4.** Differentiation on substrates in growth media. Cells were cultured on (a) flat BMG, (b) 200nm BMG in normal media without any differentiation supplements. (c) Quantification of adiponectin fluorescence (in red), (student’s t-test, n=3 samples, at least 5 images per sample, not significant, scale bar = 100 μm) DAPI was used for nuclear staining (in blue). Error bars represent standard error mean (SEM).


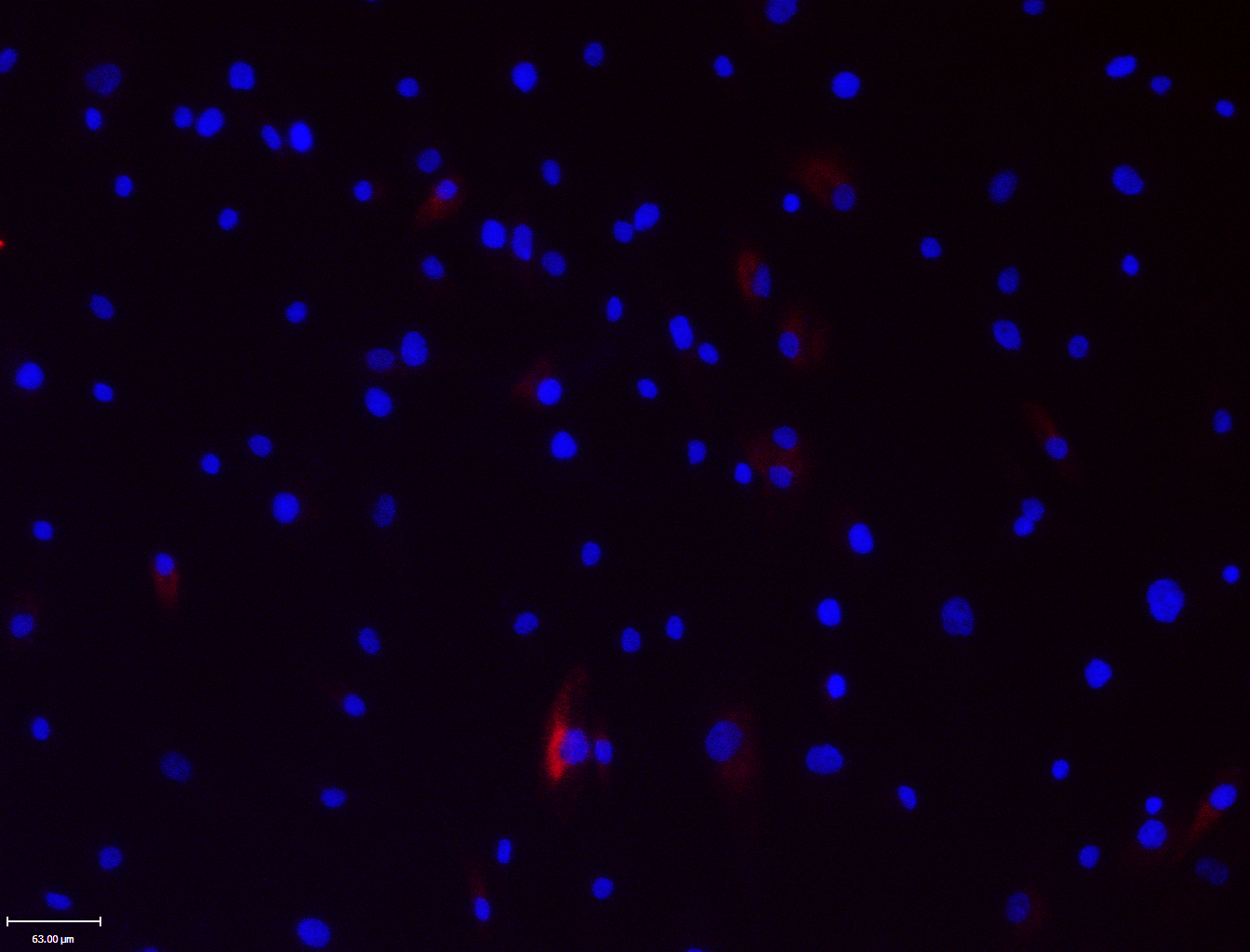

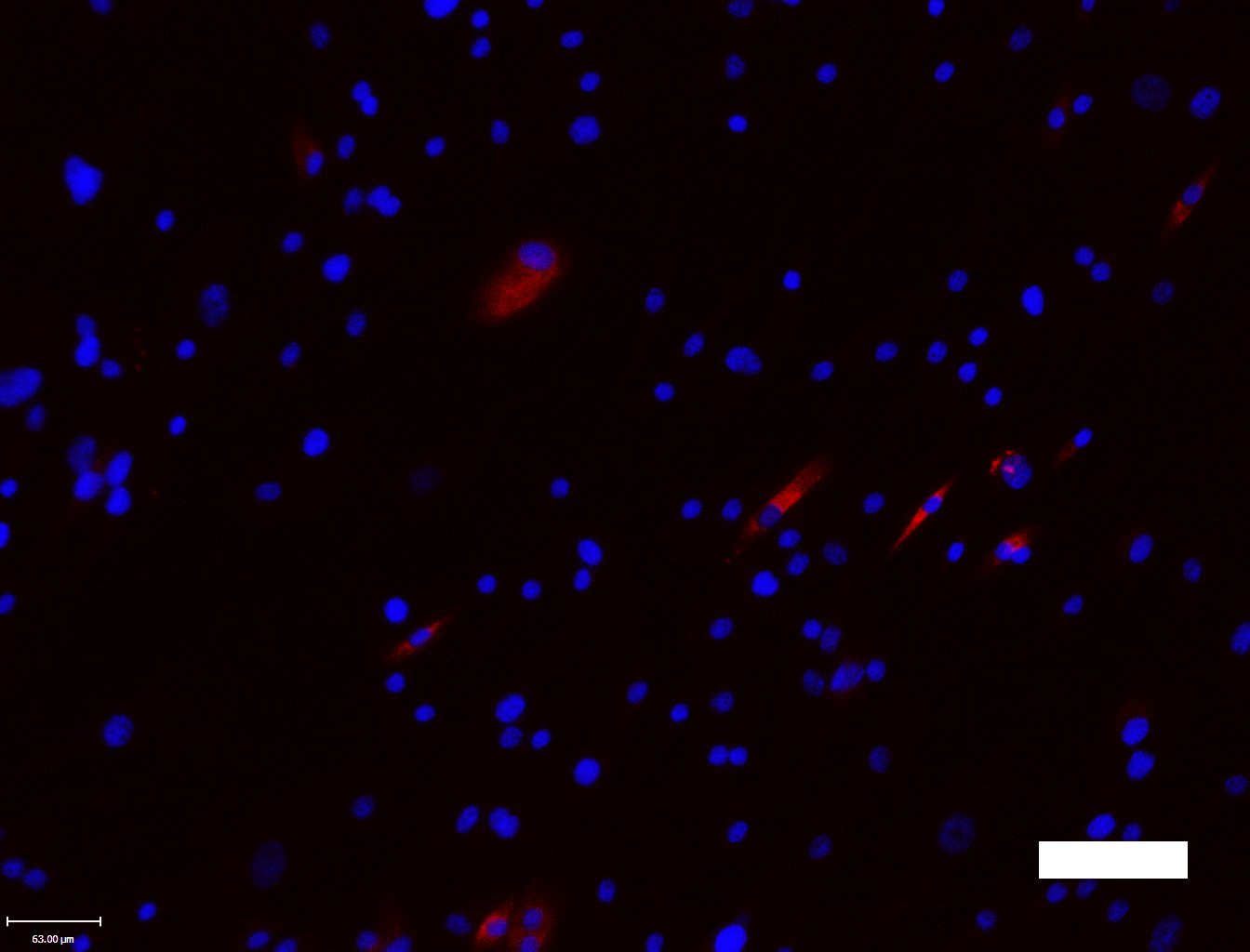


**a**

**b**

**c**
